# 2670. Sexual Health Evaluation Among People Tested for Mpox in an Urban Health System

**DOI:** 10.1093/ofid/ofad500.2281

**Published:** 2023-11-27

**Authors:** Joyce Jones, William Garneau, Gaby Dashler, Nathan Kwon, Elizabeth Gilliams, Matthew M Hamill, David Rudolph, Jeanne Keruly, Eili Klein, Bhakti Hansoti, Kelly Gebo

**Affiliations:** Johns Hopkins University School of Medicine, Baltimore, Maryland; Johns Hopkins University School of Medicine, Baltimore, Maryland; Johns Hopkins School of Medicine, Baltimore, Maryland; Johns Hopkins University School of Medicine, Baltimore, Maryland; Johns Hopkins University School of Medicine, Baltimore, Maryland; Johns Hopkins University School of Medicine, Baltimore, Maryland; Johns Hopkins University School of Medicine, Baltimore, Maryland; The Johns Hopkins University School of Medicine, Baltimore, MD; Johns Hopkins School of Medicine, Baltimore, Maryland; Department of Emergency Medicine, Johns Hopkins University, Baltimore MD, Baltimore, Maryland; Johns Hopkins, Baltimore, MD

## Abstract

**Background:**

The predominant mode of transmission in the 2022 mpox outbreak was sexually associated. Best practices for mpox evaluation include a sexual history, an anal and/or genital exam and STI testing. We hypothesized that the quality of clinical evaluation (QoC) for mpox varied by clinical setting.

**Methods:**

A retrospective chart review was conducted on all patients in the Johns Hopkins Health System (JHHS) who tested for mpox from June 15 through December 15, 2022. Outcome measures included three QoC metrics (record of sexual history, anal and/or genital exam and concomitant STI testing). Fisher’s exact test was used to compare the frequency of QoC indicators by location of mpox testing (emergency department/urgent care [ED/UC], HIV/sexual health clinic [HIV/SH] or non-HIV/SH outpatient clinic [non-HIV/SH]).

**Results:**

Of 269 patients tested for mpox, median age was 37 years, 48% were Black, 8% Latino, 71% assigned male sex at birth, 2% transgender women and 25% living with HIV (Table 1). Location of mpox testing was ED/UC (n=165), HIV/SH (n=50), or non-HIV/SH (n=54). Rates of mpox diagnosis and documentation of a sexual history, anal and/or genital exam and concomitant STI testing varied by site and were statistically significantly different (Table 2). HIV/SH had the highest rate of mpox test positivity (58%) and were most likely to meet these QoC indicators (96%, 78% and 82%, respectively) followed by ED/UC (74%, 50%, 57%, respectively) and HIV/SH clinics (68%, 32%, 30%).

**Figure:**
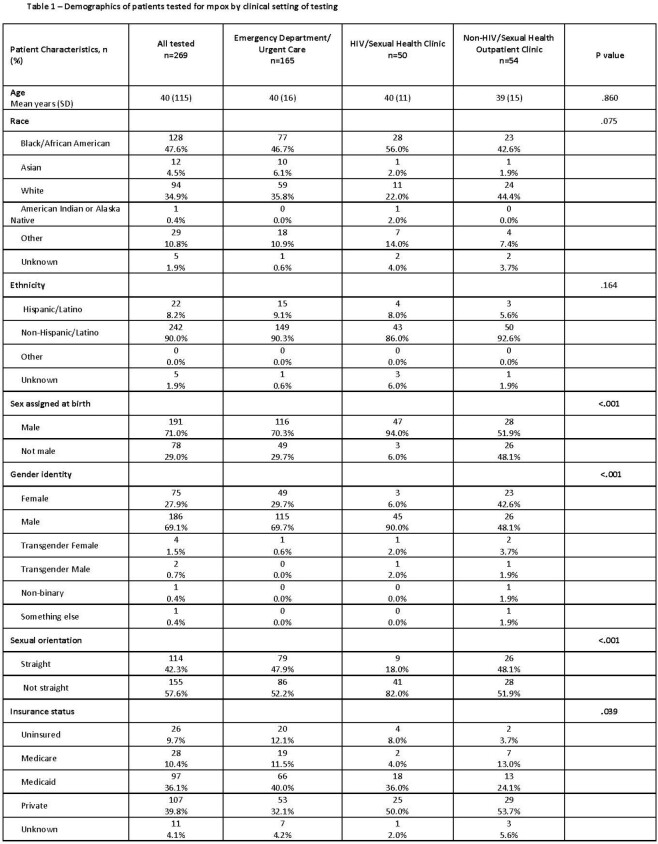

**Figure:**
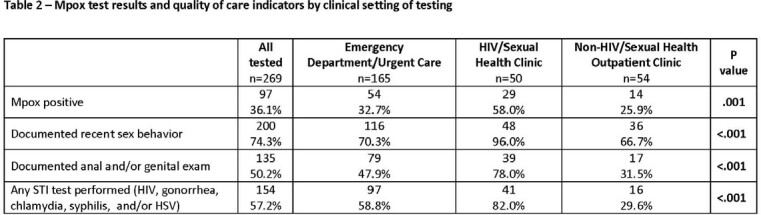

**Conclusion:**

In a high-volume tertiary care health system, only 50% of individuals tested for mpox had a documented anal and/or genital exam and 57% had concomitant STI testing. QoC indicators for mpox evaluation were highest in clinics where sexual health assessments are routine (HIV/sexual health), intermediate in ED/urgent care settings and lowest in non-HIV/sexual health settings. These findings support other studies that demonstrate a lack of consistent sexual health evaluation among individuals at risk for STIs. Strategies that decrease barriers to completed sexual history, exam and STI testing should be explored including audio-computer assisted patient report of sexual history, patient self-collection of STI testing and provider training.

**Disclosures:**

**Matthew M. Hamill, MBChB, PhD**, Roche diagnostics: Honoraria **Kelly Gebo, MD, MPH**, Pfizer: Advisor/Consultant|Spark HealthCare: Advisor/Consultant

